# Bisoprolol Transdermal Patch Is Effective for the Treatment of AF Tachycardia

**DOI:** 10.1155/2018/9139302

**Published:** 2018-05-21

**Authors:** Yoh Arita, Hajime Saeki, Miwa Miyoshi, Shinji Hasegawa

**Affiliations:** Department of Cardiology, Japan Community Healthcare Organization (JCHO) Osaka Hospital, 4-2-78 Fukushima, Fukushima-ku, Osaka 553-0003, Japan

## Abstract

Atrial fibrillation (AF) is an irregular and often rapid heart rate that can increase the risk of stroke, heart failure, and other heart-related complications. Its incidence increases with age and the presence of concomitant heart disease. We present the cases of a 93-year-old woman, an 82-year-old man, and an 87-year-old woman who developed AF tachycardia. This report highlights the use of a bisoprolol transdermal patch to treat AF tachycardia in 3 adult elderly patients. In this paper, we report an initial treatment strategy using a bisoprolol transdermal patch and show heart rate trends for 24 hours.

## 1. Introduction

Clinical atrial fibrillation (AF) is associated with increased rates of stroke, heart failure, mortality, hospitalization, and cognitive decline, much of which may present suddenly and constitute irretrievable harm [1, 2]. AF symptoms often include heart palpitations, shortness of breath, and weakness. Rate control is possible in the majority of patients with AF. Beta- (*β*-) blockers have been the most effective drugs [3]. However, swallowing tablets or capsules is sometimes difficult for elderly people because of dysphagia [4]. Moreover, aspiration pneumonia can be associated with dysphagia [5]. Bisoprolol is also available as a transdermal patch in Japan. Medication adherence is better with the use of a transdermal patch than with the use of tablets, particularly in elderly patients who might have difficulty with oral administration. In this paper, we report an initial treatment strategy for AF tachycardia using a bisoprolol transdermal patch in elderly patients.

## 2. Case Presentation

### 2.1. Case 1

**Figure 1:**
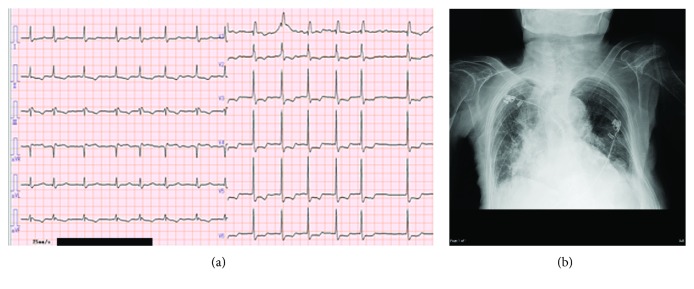
(a) Electrocardiogram (ECG, case 1) findings upon admission. ECG showing atrial fibrillation (AF) and a complete right bundle branch block. (b) Chest X-ray (CXR) findings during AF tachycardia. CXR showing severe pulmonary edema and congestion.

**Figure 2:**
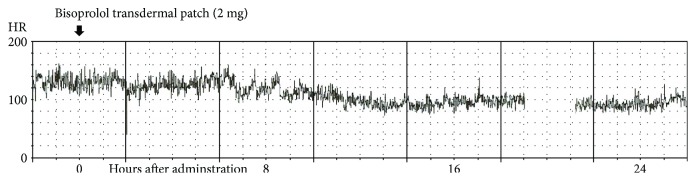
Patient (case 1) heart rate (HR) trends during treatment. The gap in the record is due to battery exhaustion.

This 93-year-old woman, a resident of a special elderly care nursing home, was referred to our hospital for the treatment of cellulitis. She related a history of treatment for hypertension and atrial fibrillation (AF). Upon arrival, her blood pressure (BP) was 119/83 mmHg and heart rate (HR) was 82 bpm. An electrocardiogram (ECG) demonstrated AF and a complete right bundle branch block (Figure 1(a)). During the treatment for cellulitis using antibiotics, she complained of dyspnea. Her HR increased to 140 bpm and her chest X-ray (CXR) showed pulmonary edema and congestion (Figure 1(b)). Echocardiography demonstrated preserved cardiac contractility with an ejection fraction (EF) of 60%, indicating heart failure with a preserved EF. She was administered furosemide (20 mg/day) to treat heart failure. In addition, a bisoprolol transdermal patch (2 mg) was applied to her chest. Her HR trends were significantly decreased within 8 hours and the control of HR continued for 24 hours (Figure 2). Eventually, her CXR and symptoms improved.

### 2.2. Case 2

**Figure 3:**
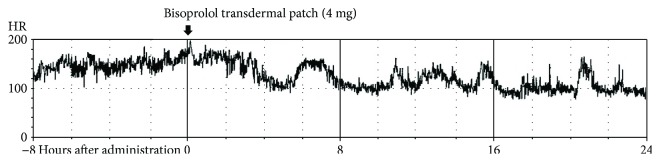
Patient (case 2) heart rate (HR) trends during treatment.

An 82-year-old man was admitted to our hospital for the treatment of ileus. He related a history of treatment for persistent AF and hypertension. He was administered bisoprolol fumarate tablets (2.5 mg/day) for AF before admission. His HR on admission was 87 bpm. However, he could not take oral medicine because of fasting for treatment of ileus. After 3 days of fasting, he developed AF tachycardia and his HR increased to 150 bpm. Bisoprolol transdermal patch (4 mg) was applied to his chest. This dose was equal to a 2.5 mg bisoprolol fumarate tablet. His HR trends were significantly decreased after 8 hours of bisoprolol transdermal patch, and the frequency and duration of AF decreased until 24 hours after administration (Figure 3).

### 2.3. Case 3

**Figure 4:**
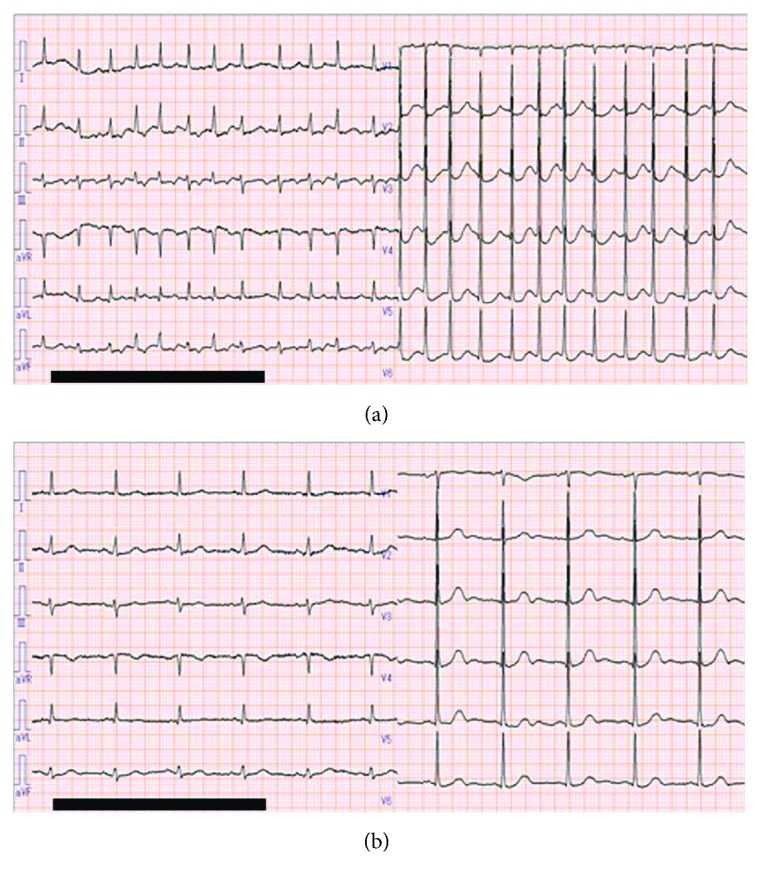
Electrocardiogram (ECG, case 3) findings upon admission. (a) ECG showing atrial fibrillation (AF). (b) ECG showing sinus rhythm (SR). The heart rhythm often spontaneously alternated between AF and SR.

**Figure 5:**
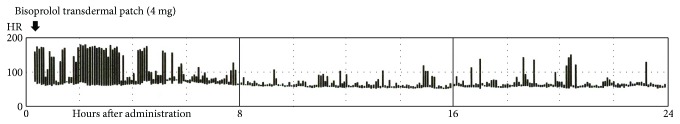
Patient (case 3) heart rate (HR) trends during treatment.

An 87-year-old woman was referred to our hospital for treatment of AF with palpitation and dyspnea (Figure 4(a)). She related a history of treatment for hypertension and cerebral infarction but no history of heart failure and/or arrhythmia. Upon arrival, her BP was 102/54 mmHg and HR was 151 bpm. Her heart rhythm often spontaneously alternated between AF and sinus rhythm (Figure 4(b)). Echocardiography demonstrated preserved cardiac contractility with an EF of 64%, indicating heart failure with preserved EF. She was administered verapamil (5 mg) injection; however, there was no decrease in HR or frequency of rhythm alternation. Next, bisoprolol transdermal patch (4 mg) was applied to her chest. Her HR trends were significantly decreased after 6 hours on the bisoprolol transdermal patch and the frequency and duration of AF decreased until 24 hours after administration (Figure 5). Moreover, her symptoms were improved.

## 3. Discussion

AF is an irregular and often rapid heart rate that can increase the risk of stroke, heart failure, and other heart-related complications. Its incidence increases with age and the presence of concomitant heart disease [6]. Rate control in AF improves the quality of life, reduces morbidity, and decreases the potential for tachycardia-induced cardiomyopathy. Multiple agents, including *β*-blockers, nondihydropyridine calcium channel blockers, digoxin, and certain antiarrhythmic drugs, including amiodarone and sotalol, have been evaluated for efficacy in attaining rate control [1]. *β*-blockers are the most commonly used drugs to control the ventricular rate during AF [3]. *β*-blockers also have an antiarrhythmic effect due to the suppression of sympathetic activity [7]. By reducing sympathetic tone, conduction over the atrioventricular node is slowed and atrioventricular nodal refractoriness is increased.

Bisoprolol, also available as a transdermal patch in Japan, is indicated for the management of hypertension [8]. However, bisoprolol fumarate tablets are used for the management of patients with AF tachycardia [6]. Switching therapy from landiolol to bisoprolol transdermal patch is often performed in patients with AF tachycardia because the latter is relatively easy to manage [9–11]. A recent study reported efficacy and safety when switching from bisoprolol fumarate tablets to a bisoprolol transdermal patch at a dose conversion rate of 5 : 8 [12]. There are several benefits to using a transdermal patch instead of tablets. First, medication adherence is better because patients or caregivers can monitor medication through direct observation of the transdermal patch. Second, a transdermal patch can be used in patients for whom oral treatment is difficult, including those whose swallowing function has been impaired, those with gastrointestinal disease, and those who require tracheal intubation for an operation or pneumonia.

The current cases may provide physicians with an initial treatment strategy for the use of a bisoprolol transdermal patch for AF tachycardia patients and especially in elderly patients who are relatively difficult to treat with oral medication.
